# PSInet: a new global water potential network

**DOI:** 10.1093/treephys/tpae110

**Published:** 2024-08-27

**Authors:** Ana Maria Restrepo-Acevedo, Jessica S Guo, Steven A Kannenberg, Michael C Benson, Daniel Beverly, Renata Diaz, William R L Anderegg, Daniel M Johnson, George Koch, Alexandra G Konings, Lauren E L Lowman, Jordi Martínez-Vilalta, Rafael Poyatos, H Jochen Schenk, Ashley M Matheny, Katherine A McCulloh, Jesse B Nippert, Rafael S Oliveira, Kimberly Novick

**Affiliations:** O'Neill School of Public & Environmental Affairs, Indiana University Bloomington, 702 N Walnut Grove St, Bloomington, IN 47405, USA; Department of Biology, West Virginia University, Morgantown, VA 26506, USA; Arizona Experiment Station, University of Arizona, 1140 E. South Campus Dr., Tucson, AZ 85721, USA; Department of Biology, West Virginia University, Morgantown, VA 26506, USA; O'Neill School of Public & Environmental Affairs, Indiana University Bloomington, 702 N Walnut Grove St, Bloomington, IN 47405, USA; O'Neill School of Public & Environmental Affairs, Indiana University Bloomington, 702 N Walnut Grove St, Bloomington, IN 47405, USA; Arizona Experiment Station, University of Arizona, 1140 E. South Campus Dr., Tucson, AZ 85721, USA; School of Biological Sciences and Wilkes Center for Climate Science and Policy, University of Utah, Salt Lake City, UT 84112, USA; Warnell School of Forestry and Natural Resources, University of Georgia, Athens, GA 30602, USA; Center for Ecosystem Science and Society & Department of Biological Sciences, Northern Arizona University, Flagstaff, AZ 86011, USA; Department of Earth System Science, Stanford University, Stanford, CA 94305, USA; Department of Engineering, Wake Forest University, Winston-Salem, NC 27101, USA; CREAF, E08193 Bellaterra (Cerdanyola del Vallès), Catalonia, Spain; Universitat Autònoma de Barcelona, E08193 Bellaterra (Cerdanyola del Vallès), Catalonia, Spain; CREAF, E08193 Bellaterra (Cerdanyola del Vallès), Catalonia, Spain; Universitat Autònoma de Barcelona, E08193 Bellaterra (Cerdanyola del Vallès), Catalonia, Spain; Department of Biological Science, California State University, Fullerton, CA 92831, USA; Department of Earth and Planetary Sciences, Jackson School of Geological Sciences, University of Texas at Austin, Austin, TX 98705, USA; Department of Botany, University of Wisconsin-Madison, Madison, WI 53706, USA; Division of Biology, Kansas State University, Manhattan, KA 66506, USA; Department of Plant Biology, University of Campinas (UNICAMP), Campinas, SP, Brazil; O'Neill School of Public & Environmental Affairs, Indiana University Bloomington, 702 N Walnut Grove St, Bloomington, IN 47405, USA

**Keywords:** database, drought, network, plants, plant hydraulics, water potential

## Abstract

Given the pressing challenges posed by climate change, it is crucial to develop a deeper understanding of the impacts of escalating drought and heat stress on terrestrial ecosystems and the vital services they offer. Soil and plant water potential play a pivotal role in governing the dynamics of water within ecosystems and exert direct control over plant function and mortality risk during periods of ecological stress. However, existing observations of water potential suffer from significant limitations, including their sporadic and discontinuous nature, inconsistent representation of relevant spatio-temporal scales and numerous methodological challenges. These limitations hinder the comprehensive and synthetic research needed to enhance our conceptual understanding and predictive models of plant function and survival under limited moisture availability. In this article, we present PSInet (PSI—for the Greek letter Ψ used to denote water potential), a novel collaborative network of researchers and data, designed to bridge the current critical information gap in water potential data. The primary objectives of PSInet are as follows. (i) Establishing the first openly accessible global database for time series of plant and soil water potential measurements, while providing important linkages with other relevant observation networks. (ii) Fostering an inclusive and diverse collaborative environment for all scientists studying water potential in various stages of their careers. (iii) Standardizing methodologies, processing and interpretation of water potential data through the engagement of a global community of scientists, facilitated by the dissemination of standardized protocols, best practices and early career training opportunities. (iv) Facilitating the use of the PSInet database for synthesizing knowledge and addressing prominent gaps in our understanding of plants’ physiological responses to various environmental stressors. The PSInet initiative is integral to meeting the fundamental research challenge of discerning which plant species will thrive and which will be vulnerable in a world undergoing rapid warming and increasing aridification.

## Water potential data are crucial for understanding plant responses to changing environmental conditions

Ecosystem function is strongly controlled by water potential (Ψ) gradients from soil to plants and to the atmosphere. In many ways, Ψ can be imagined as the ‘blood pressure’ of the ecosystem; in the same way that blood pressure is a key measure of human health, Ψ is a key indicator of plant performance. Gradients in Ψ—within the soil, between plant roots and leaves, and between leaves and the atmosphere—are the energetic basis for ecosystem water fluxes. Leaf water potential (Ψ_L_) directly controls stomatal conductance and photosynthesis (Jarvis 1976; Sperry 2000) and is coupled with branch and stem water potential (Ψ_X_), which determine the risk of drought-driven hydraulic failure (Choat et al. 2012). Severely limited access to soil moisture can cause detrimental declines in plant Ψ_L_ and Ψ_X_, which can in turn induce stomatal closure, cause reductions in photosynthesis and growth, propagate embolism through the xylem network and limit water transport. Consequently, Ψ is a first-order control on how much carbon ecosystems remove from the atmosphere, how much water they move to the atmosphere in the process and the likelihood that plants survive droughts. Over the past decade, there has been a surge of interest in uncovering the relationships between Ψ and physiological traits (Martínez-Vilalta et al. 2017; McCulloh et al. 2019; Li et al. 2020; Flo et al. 2021; Kannenberg et al. 2021), incorporating plant hydraulics into predictive models (Kennedy et al. 2019; Mirfenderesgi et al. 2016; Sperry et al. 2017; Li et al. 2020) and advancing diverse remote-sensing approaches for detecting Ψ (Momen et al. 2017; Konings et al. 2019, 2021).

**Figure 1:**
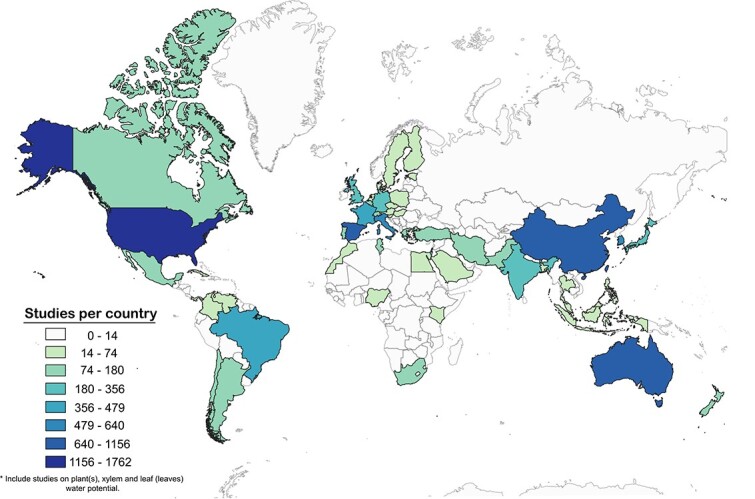
Geographic distribution of studies on plant water potential for both natural and agricultural ecosystems from 1970 to 2023 (including plants, leaves and xylem). Data from a Scopus search of literature (plant[s] water potential’ OR ‘xylem water potential’ OR ‘leaf water potential’ OR ‘stem water potential’ in title, abstract or keywords) and visualized by color-coding the number of studies in each country. Notably, the USA stands out with the highest number of studies (1257), followed by China (794) and Australia and Spain (507 each). There is a pronounced underrepresentation in regions such as central and South America, Africa and eastern European countries. These areas exhibit a significant gap in research on Ψ, highlighting the need for more comprehensive global coverage in the field.

However, while our understanding of plant Ψ is theory-rich, it is currently data-poor and there exist significant challenges in its study. Despite the abundance of time series data collected in some regions, accessibility remains a considerable hurdle due to the absence of a centralized database. Additionally, published Ψ studies tend to be biased towards ecosystems within North America (USA and Canada) and Europe (Fig. 1), which together comprise ~47% of studies conducted globally even though these regions represent only 24% of the global land area. A major challenge in studying Ψ lies in the absence of a centralized repository that could facilitate the synthesis of essential knowledge and bridge prominent gaps in our comprehension of plants’ physiological responses to diverse environmental stressors. The absence of a unified information source, coupled with geographical biases, plays a pivotal role in conspicuously underrepresenting critical ecosystems globally. Furthermore, this deficiency in Ψ data deprives the scientific community of indispensable insights necessary for a holistic comprehension of Earth’s interlinked systems and their responses to environmental dynamics.

## Plant water potential measurements: status and future needs

The predominant approach for assessing plant Ψ_L_ and Ψ_X_ currently involves manual measurements using a Scholander-style ‘pressure chamber’ (Scholander et al. 1965; Rodriguez-Dominguez et al. 2022). These measurements provide estimates of plant Ψ_L_ and Ψ_X_ under specific conditions at a specific moment in time. However, for a more comprehensive understanding of a plant's water stress, it is essential to collect data multiple times during the day and at intervals spanning weeks or longer, to capture gradients in key environmental drivers. While pressure chamber data are temporally discrete, these data are usually collected twice daily (e.g. and pre-dawn and mid-day), often for several weeks or months. Thus, a rich global database would be particularly useful to comprehend Ψ at diurnal timescales and to capture seasonal dynamics and fluctuations in soil moisture. It aids in evaluating the water status and drought responses of vegetation within natural ecosystems. Chamber Ψ can be monitored to optimize water management practices in agriculture and horticulture (Bittelli 2010; Levin and Nackley 2021). Finally, it serves as a reliable reference dataset for the validation of remote sensing techniques used in monitoring vegetation water status (Momen et al. 2017; Holtzman et al. 2021).

Records of pre-dawn and mid-day water potential collected with pressure chambers at weekly (or longer) timescales may be sufficient to link Ψ_L_ and Ψ_X_ dynamics to variations in soil water availability within a specific study. However, the time-intensive nature of this sampling approach usually limits the length of these time series. Furthermore, the time intervals at which most pressure chamber data are gathered are not sufficiently fine to capture more rapid sub-diurnal processes, such as stomatal response to changes in vapor pressure deficit (VPD; Novick et al. 2022) and daily fluctuations in plant water storage (Matheny et al. 2017). Moreover, collecting Ψ_L_ and Ψ_X_ data involves conducting field work, which presents unique inherent challenges.

## The PSInet water potential dataset and community

The PSInet Research Coordination Network (https://psinetrcn.github.io/) is a new centralized global dataset of plant and soil water potential measurements that will confront the Ψ information gap and enable the pursuit of previously intractable questions about plant responses to environmental drivers. PSInet will function as a bridge connecting readily available information about environmental variables and eco-physiological responses from other network databases. The latter include continuous flux tower observations of ecosystem-scale carbon and water fluxes (e.g. AmeriFlux and FLUXNET; Baldocchi 2008; Novick et al. 2018), the SAPFLUXNET database of continuous tree water-use observations (Poyatos et al. 2012) and the Xylem Functional Traits database (Choat et al. 2012), which is the primary source of information about plant hydraulic traits within the larger TRY plant traits database (Kattge et al. 2019). While these networks aggregate many important eco-physiological variables and traits, they do not provide the time series of Ψ that are required to mechanistically link environmental drivers and physiological responses, and to benchmark and inform modeling and remote-sensing approaches. This is the gap that PSInet will fill, to accelerate our theoretical and predictive understanding of plant–environment responses, now and for a warmer future.

**Figure 2:**
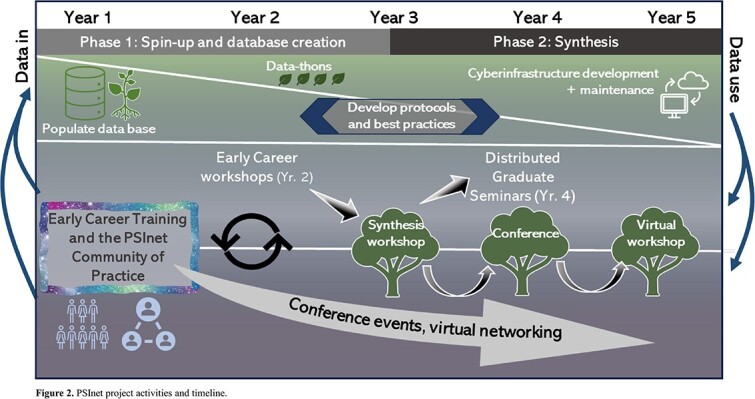
PSInet project activities and timeline.

Importantly, PSInet is not just a network of data but a network of people, organized around coordinated research, training and community-building activities designed to increase the availability, integrity and accessibility of Ψ information to a diverse scientific community. An overarching goal of PSInet is to create a Community of Practice with greater gender balance, racial diversity and geographic diversity than the status quo. We foster a diverse and inclusive network environment with multiple mechanisms to advance the careers of demographically, geographically and intellectually diverse cohorts of early career scientists. Within the scope of PSInet, we will implement multiple mechanisms to support the training of the next generation of ecophysiologists, including multiple early career summer workshops such as Phys-Fest, a forthcoming early career workshop on plant hydraulics, a forthcoming distributed graduate seminar, and numerous opportunities to participate in virtual and in-person workshops, conference sessions and seminars (Fig. 2). Implicit in all PSInet Community of Practice activities is an emphasis on elevating the work and careers of scientists from underrepresented demographics and geographies.

**Figure 3:**
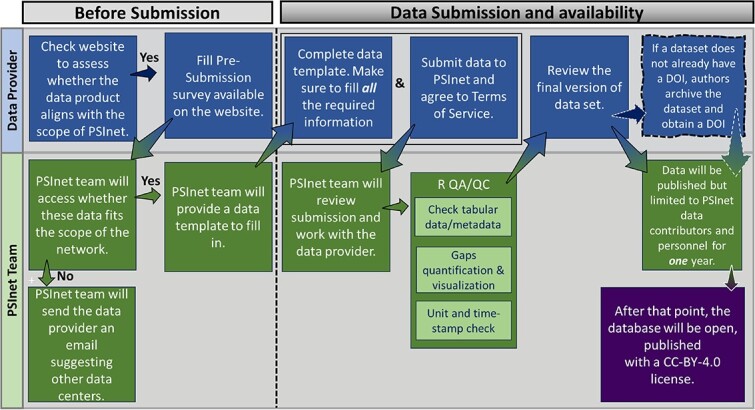
PSInet data flow from submission to publication. The first step is completing the pre-submission survey available on the PSInet website (https://psinetrcn.github.io/submit.html). Subsequently, the contributor prepares the data for submission, after which PSInet personnel conduct quality assurance and quality control (QA/QC) checks. Data contributors are then responsible for final approval and the assignment of a unique data identifier (DOI). The data become accessible initially to the contributors and afterwards to the public.

In early 2024, we initiated collection of plant water potential data and invite potential data contributors to join the effort. As a benefit to contributing data for free and open dissemination via PSInet, data contributors will receive priority access to the PSInet data for an embargo period of 1 year and opportunities to participate in PSInet networking, career development, and collaborative activities. Up to two contributors associated with each dataset contributed to the PSInet database will have the opportunity to collaborate on a forthcoming data paper. More information about the PSInet data submission process is available in Fig. 3 and at https://psinetrcn.github.io/submit.html. We are also actively seeking volunteer participation in the organization and execution of PSInet networking and outreach activities. Interested participants can indicate their interest by visiting https://psinetrcn.github.io/join.html. Our initial focus is on collecting plant water potential data and associated ancillary measurements. In the future, we envision an extension of PSInet to collect and aggregate information on soil water potential from sites that do not necessarily monitor plant water potential.

## Alternative techniques for measuring Ψ

**Figure 4:**
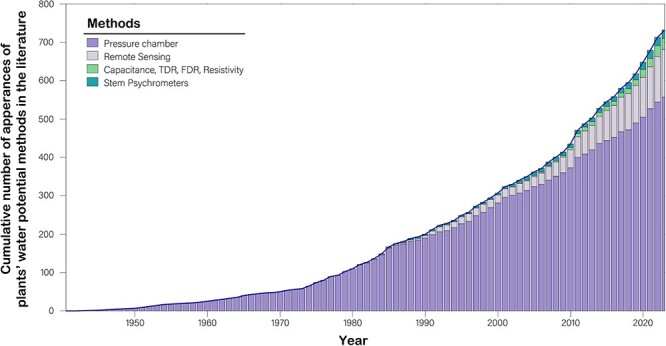
Cumulative count of appearances of different direct and indirect methods for estimating plant water potential in a Scopus search of literature (plant[s] water potential’ OR ‘xylem water potential’ OR ‘leaf water potential’ OR ‘stem water potential’ in title, abstract or keywords). Note that counts represent individual appearances of each method, not papers (e.g. a paper can have multiple methods). We found that the pressure chamber method (e.g. Scholander et al. 1965) is historically the most popular (~87%) followed by remote sensing techniques including methodological developments and estimations of plant Ψ (~10%). However, in the last 10 years, the popularity of the different methods has been changing. The pressure chamber method remains the most popular with ~79%, followed by remote sensing (~15%), geophysical techniques such as resistivity, TDR, FDR (~2.7%) and psychrometry (2.6%).

Over the past three decades, there has been considerable progress in the development of alternative techniques for monitoring Ψ_L_ and Ψ_X_ and plant's water status to address the discontinuous and discrete nature of pressure chamber Ψ measurements (Fig. 4). Several techniques offer promising, automated methods to monitor Ψ on the order of days to months. These techniques could be broadly classified as (i) direct sensing of water potential such as psychrometry, and most recently micro-tensiometers and hydrogel nano-reporters, and (ii) indirect measurements such as remote sensing, or geophysical monitoring methods (e.g. capacitance such as time domain reflectometry [TDR], frequency domain reflectometry [FDR] and electrical resistivity). As a network of data and people involved in water potential, PSInet is well-poised to evaluate Ψ data generated with newer techniques, facilitate intercomparisons across methodologies, and promote best practices for collecting and analyzing these data.

These techniques allow estimations and measurements of plant Ψ at timescales that can capture high frequency or large spatial dynamics, and which complement the scales over which water and carbon fluxes are often measured and modeled. However, their practical implementation remains limited due to acknowledged constraints associated with these methods. Overall, the limitations associated with these techniques challenge our ability to synthesize and interpret the water potential ‘observations’. Factors include: (i) assessing method selection based on the specific plant tissue under investigation (e.g. Ψ_L_ vs Ψ_X_ vs root water potential—Ψ_R_), (ii) scaling challenges from individual plants to the ecosystem level, (iii) the essential but often problematic tasks of instrument maintenance under field conditions (e.g. accessing canopies and the necessity for routine checking due to tree protective mechanisms), (iv) the necessity of species-specific calibration parameters, and (v) potential biases stemming from the sensitivity of instruments to environmental variables. Collectively, these techniques represent valuable resources for bridging the spatial and temporal gaps inherent to pressure chamber data, but we urgently need openly accessible databases and community crafted best practices to overcome these operational difficulties.

For instance, remote sensing, with its potential for broad spatial coverage, appears as the second most common technique used to study and provide information about Ψ (Fig. 2). Several relevant approaches exist, including hyperspectral, L-band, thermal and microwave measurement. Among these methods, microwave remote sensing, as highlighted by Konings et al. (2021), shows promise since it can penetrate clouds and is sensitive to vegetation water content. However, this approach is not currently sufficiently mature to be used for estimation of Ψ without extensive ground calibration and validation data. Furthermore, a substantial portion of the current studies on Ψ utilizing remote sensing techniques tends to focus more on evaluating various methodologies rather than fundamental water potential research. Over the past few decades, alternative techniques like capacitance sensors (TDR, FDR—Matheny et al. 2017), electrical resistivity (Cardenas et al. 2014), hydrogel nanoreporters (Jain et al. 2021) and even high-resolution stem dendrometry (Drew et al. 2011; Eller et al. 2017) have emerged as suitable options for long-term, high-resolution studies across various plant types and specific tissues (particularly for Ψ_R_ and Ψ_X_)_._ However, these methods also rely on indirect measurements since they measure water content and approximate Ψ from this data (much like microwave remote sensing does). Moreover, these techniques require precise, species-specific calibration parameters that may impact measurement accuracy and limit generality to other species or ecosystems.

Stem psychrometry has been proven suitable for monitoring Ψ_X_ directly on individual plants at longer temporal resolutions (Dixon and Tyree 1984; Guo et al. 2019; Kannenberg et al. 2022), but it can present significant limitations, especially concerning the thermocouples in the sensors. High-precision Peltier-style thermocouples within the stem sensor can become occluded due to the plant wounding response, with the severity of this response varying significantly among different species. Moreover, this technique relies on the cooling effect resulting from water evaporation, which can be sensitive to daily and seasonal temperature and humidity fluctuations in natural conditions. To mitigate these limitations, careful calibration and frequent maintenance, as well as strong insulation and shielding to limit temperature gradients, are imperative. Furthermore, data must be corrected to account for temperature-related errors (Quick et al. 2018).

More recently, microtensiometers (Pagay et al. 2014; Pagay 2021; Dainese et al. 2021, 2022; Lakso et al. 2022; Conesa et al. 2023) have emerged as valuable tools for continuously monitoring plant water potential (Ψ) directly at a finer scale. It stands out that microtensiometers offer high-resolution measurements of 0.1 bar with measurements every 20 min. However, it is important to note that, owing to their small-scale nature, both microtensiometers and psychrometers provide localized measurements that may not be reflective of whole-plant dynamics. Achieving a comprehensive understanding of plant water potential may need the use of multiple devices, adding complexity to the study. Additionally, regular maintenance may be required to ensure the continued accuracy and reliability of microtensiometer measurements due to cavitation of water in the sensing system.

We recognize that the challenges discussed are not exclusive to monitoring plant Ψ. For instance, measurements of soil water potential (Ψ_S_), which dictates water availability to plant roots, encounter similar hurdles (Martínez-Vilalta et al. 2021; Khare et al. 2022; Novick et al. 2022). Current soil sensors often have limitations, typically providing accuracy only down to −2 MPa (with a few exceptions like the dielectric now available as TEROS 21 from METER). Additionally, the construction of accurate water retention curves, enabling the conversion of water content to water potential, can be intricate and demanding.

For these reasons, another important objective of PSInet is to facilitate the creation of community-developed best practices and protocols for emerging approaches to measuring water potential along the soil–plant–atmosphere continuum. The diversity of techniques used to measure Ψ emphasizes the necessity for inter-comparison and integration, aiming to streamline sensor choices in future studies. This juncture presents an opportune moment for a renewed emphasis on field data collection and the establishment of new networks, such as PSInet, for aggregating observations across various sites. Coupled with innovative approaches for integrating these observations into Earth system models, such initiatives can significantly advance our understanding of the intricate interplay within the soil–plant–atmosphere continuum.

## Scientific questions answerable using data from PSInet

We anticipate that the extensive data and collaborative ethos of PSInet will be instrumental in addressing a wide range of crucial research questions spanning plant-to-ecosystem scales. These questions may include topics such as the following.

### How do plants respond to increasing VPD induced by climate change?

Plants independently and interactively respond to water deficits both in the soil (e.g. soil water potential, Ψ_S_) and the air (determined by VPD). Climate change is driving substantial increases in VPD almost everywhere (Ficklin and Novick 2017; Grossiord et al. 2020), but the directionality of soil moisture projections varies, increasing in some regions and decreasing in others (Cook et al. 2015). Consequently, the relationship between Ψ_S_ and VPD is changing, and understanding how plants respond to each factor is essential for making reliable projections about plant function and survival in the future. Generalizing the role of VPD in governing plant dynamics requires plant Ψ time series collected at diurnal timescales over which VPD varies significantly, but soil moisture does not, necessary to disentangle the relative contribution of soil versus atmospheric drought. Continuous plant Ψ data are especially well-suited for this challenge, though diurnal pressure chamber data are also useful (Koch et al. 2015; Guo and Ogle 2018; Gersony et al. 2020).

### What are the mechanisms underlying drought-induced plant mortality and hydraulic failure?

There is broad consensus that hydraulic failure, or the cessation of xylem water transport due to embolism, triggers drought-induced mortality in plants (Adams et al. 2017; Choat et al. 2018; Hammond et al. 2019; McDowell et al. 2022). The risk of hydraulic failure is typically assessed using the hydraulic safety margin (HSM), quantified as the difference between the minimum plant Ψ experienced by the plant and a measure of embolism resistance (e.g. P50, the Ψ causing 50% loss of hydraulic conductivity, Meinzer et al. 2009; Choat et al. 2012). In other words, HSM = minimum plant Ψ—P50. HSM integrates a measure of absolute stress tolerance determined in the laboratory (P50) with a measure of extreme exposure at the tissue level, yielding a promising indicator of mortality risk (Anderegg et al. 2016; Benito Garzón et al. 2018; Venturas et al. 2020). However, determining minimum Ψ is methodologically challenging, and current estimates are known to be biased due to the significant effect of sample size on absolute extremes (Martínez-Vilalta et al. 2021). PSInet will improve the quality and quantity of Ψ data available to assess drought stress exposure in plants.

### What can nocturnal water potential data reveal about pre-dawn equilibrium throughout the soil–plant–atmosphere continuum?

It is often assumed that Ψ_L_, Ψx and Ψ_S_ equilibrate during pre-dawn hours (Donovan et al. 2001; Fisher et al. 2006). This assumption has allowed eco-physiologists to use pre-dawn observations of plant water potential (Ψ) as a proxy for root-zone ΨS, circumventing the need for direct soil Ψ measurements. However, important eco-physiological processes such as nocturnal transpiration (Novick et al. 2009) and nocturnal refilling of water storage pools (Matheny et al. 2015) can prevent pre-dawn equilibrium (Bucci et al. 2005; Caird et al. 2007; Dawson et al. 2007). Understanding what drives disequilibrium is crucial, as it lowers pre-dawn Ψ_L_ and complicates assessments of species-specific rooting depths. Continuous plant Ψ data will be a valuable source of insight because equilibrium should be evident in the stationarity of pre-dawn plant Ψ time series.

### How can we improve model predictions including plant hydraulics?

Feedback mechanisms linked to increasing drought frequency and intensity are a major source of uncertainty in land surface models (Reichstein et al. 2013, Mencuccini et al. 2019). Explicit representation of plant hydraulic processes can substantially reduce this uncertainty. Over the past 5–10 years, hydrologic and Earth system models have increasingly incorporated improved representations of plant hydraulic dynamics (Mackay et al. 2015; Sperry et al. 2017; Kennedy et al. 2019; Mirfenderesgi et al. 2016; De Cáceres et al. 2021; Xu and Trugman 2021). Site-level tests of these models show enhanced prediction accuracy (Eller et al. 2020; Lowman and Godoy 2020; Sabot et al. 2019). However, fundamental questions remain, such as: (i) the optimal structure of hydraulic models for accurately reflecting and predicting carbon and water balance (Sabot et al. 2022) and (ii) the best methods for parameterizing these models, whether through model-data fusion (Li et al. 2020) or parameterization schemes based on theoretical principles (Sperry et al. 2016; Sabot et al. 2019; Eller et al. 2020). Addressing these knowledge gaps requires a comprehensive database like PSInet.

### Can remotely sensed estimates of canopy water content capture plant and soil water potential across space and time?

One of the biggest challenges in studying Ψ is that this variable is difficult to measure even at the individual plant level. Moreover, to make informed decisions about the health of our ecosystems, it is imperative to explore strategies for linking Ψ to larger-scale observations derived from plot-level measurements or even from space (Novick et al. 2022).

Microwave remote sensing is among the most promising approaches for understanding Ψ dynamics at these scales (Konings et al. 2021). These microwave observations can be used to determine vegetation optical depth (VOD), which is sensitive to plant water content (Jackson and Schmugge 1991) and is related to Ψ (Momen et al. 2017; Konings et al. 2019; Holtzman et al. 2021; Humphrey and Frankenberg 2023; Yao et al. 2024). However, the exact relationship between VOD and Ψ can be influenced by various factors such as spatial and temporal resolution (VOD observations derived from satellite data), vegetation heterogeneity (Konings et al. 2019) and species-specific responses. Ground validation measurements are essential to improve the accuracy and reliability of studies on the relationship between Ψ and VOD data. Our centralized Ψ data from diverse ecosystems in PSInet will facilitate linking between Ψ measurements and these larger-scale techniques.

### How much is our understanding of plant drought responses limited by lack of information about soil water potential?

The relationship between soil water potential (Ψs) and soil moisture content (θ)—often called the ‘water retention curve’ or ‘moisture release curve’—is highly non-linear and strongly dependent on soil texture and structure (Clapp and Hornberger 1978; van Genuchten 1980). Unfortunately, in-situ observations of Ψs are scarce in ecohydrological and ecological field settings, and site-specific information on water retention curves is largely absent from environmental observation networks (Novick et al. 2022). Because θ is widely measured while Ψs is not, θ is often used as a proxy for plant-available water (Green et al. 2019; Humphrey et al. 2021; Novick et al. 2016; Stocker et al. 2018). However, Ψs is a more physiologically relevant driver and better predicts ecosystem carbon fluxes compared with θ within and across sites (Baldocchi et al. 2004; Ghezzehei et al. 2019; Novick et al. 2022). Even if Ψs data were plentiful, modeling strategies to transform Ψs into θ would be necessary to connect water balance equations with water potential-driven flows. Most land surface models rely on retention curve models parameterized with pedotransfer functions (conversion from moisture content to water potential) driven primarily by soil texture (Schaap et al. 2001). Although pedotransfer function development is an active field (Van Looy et al. 2017), most are characterized by large uncertainties that propagate through ecosystem models (Fatichi et al. 2020; Novick et al. 2022; Weihermüller et al. 2021). Site-level water retention curves and/or in-situ Ψs data, which will be part of the PSInet database, may eliminate the need to rely on pedotransfer functions for site-level simulations, allowing other sources of model uncertainty to become more discernible.

## Conclusion

Understanding which species will thrive and which will falter in a warmer and drier world is a fundamental research challenge informing many applications with societal value, including agro-ecosystem management and decisions about when and where ecosystems can be leveraged to mitigate climate change. PSInet is prepared to catalyze progress in areas that have been impacted by the scarcity of Ψ information. Moreover, our network of data and people will empower eco-physiological scientists by providing essential data, tools and a collaborative community for translational science. We aim to foster connections between research communities tackling plant responses to climate change, while fostering inclusivity and providing support to scientists in diverse regions.

## Data Availability

The data that support the findings of this study were derived from the resources available in the public domain: [https://www.scopus.com/].
